# Distribution of Oncological Diagnoses in Pediatric Patients With Febrile Neutropenia and Sepsis: Is There an Association?

**DOI:** 10.7759/cureus.91220

**Published:** 2025-08-29

**Authors:** Florin-Mihai Radulescu, Marina Ionela Nedea, Irina-Magdalena Dumitru

**Affiliations:** 1 Doctoral School of Medicine, Ovidius University of Constanța, Constanța, ROU; 2 Department of Pediatrics, "Maria Sklodowska Curie” Emergency Clinical Hospital for Children, Bucharest, ROU; 3 Department of General and Pharmaceutical Microbiology, Faculty of Pharmacy, “Carol Davila” University of Medicine and Pharmacy, Bucharest, ROU; 4 Department of Infectious Diseases, Clinical Infectious Diseases Hospital of Constanța, Constanța, ROU; 5 Department of Medicine, Academy of Romanian Scientists, Bucharest, ROU

**Keywords:** febrile neutropenia, neutropenia, pediatric oncology, pediatrics, sepsis

## Abstract

Infections are a major complication in pediatric oncology, with patients at increased risk due to chemotherapy-induced neutropenia and compromised immune status. This retrospective study, conducted between 2018 and 2022 at the "Maria Sklodowska Curie" Children's Emergency Clinical Hospital in Bucharest, evaluated 150 episodes of fever and sepsis in 94 pediatric oncology patients. The results revealed significant differences between the groups analyzed: children with central nervous system tumors had a higher frequency of sepsis (p = 0.03), while patients with leukemia and lymphoma had the longest intervals between treatment initiation and fever onset. Also, profound neutropenia was more pronounced in patients with lymphomas (median neutrophil count = 0.18, p = 0.003). These results emphasize the need for personalized monitoring of infectious risk and adaptation of prophylaxis strategies according to the type of malignancy. In conclusion, the obtained data contribute to substantiating an individualized approach in the management of fever and sepsis in pediatric oncology patients.

## Introduction

Infectious complications are a significant concern among oncological patients, who often experience multiple episodes due to their immunocompromised status. A comprehensive reporting approach that accounts for the total number of infections, rather than focusing solely on individual patients, provides a more accurate representation of the burden of infections within this population. This perspective is particularly important for identifying recurrent infections in vulnerable individuals and assessing overall infection trends [1-4]. In our cohort, however, recurrence was relatively uncommon and did not significantly alter overall infection distribution patterns.

Multiple factors influence the infection risk in cancer patients, including the severity of neutropenia, the type and duration of chemotherapy, and the implementation of prophylactic antimicrobial strategies [5,6]. Neutropenia includes not only patients with low absolute neutrophil count (ANC), but also those with functional neutropenia, defined as a qualitative neutrophil dysfunction (e.g., impaired chemotaxis, phagocytosis, and oxidative burst) secondary to chemotherapy, even in the presence of normal ANC values. These patients are similarly susceptible to infections and warrant inclusion in risk assessment models [7].

While classical neutropenia is defined by a decreased ANC (<500/mm³), there is increasing evidence that chemotherapy may impair neutrophil function even in the presence of normal ANC. This phenomenon, referred to as functional neutropenia, is clinically relevant and may confer a comparable risk of infection. Several studies have shown that neutrophil functions, such as chemotaxis, oxidative burst, and intracellular killing, remain impaired after chemotherapy, despite numerical recovery of ANC [8]. We considered this concept when evaluating cases with clinical signs of infection and recent immunosuppressive therapy.

Additionally, the growing challenge of bacterial resistance complicates infection management, underscoring the need for continuous reassessment of antibiotic protocols [9].

Given these complexities, a dynamic and data-driven approach to infection surveillance is essential for optimizing patient outcomes. Real-time monitoring of infection incidence, early detection of emerging resistance patterns, and the integration of precision medicine strategies can enable more targeted antimicrobial therapies [10,11]. This article explores the impact of these factors on infection management in oncological patients and highlights the importance of adaptive strategies in improving clinical care [12].

A more nuanced understanding of how different cancer types influence infection susceptibility is crucial for optimizing risk stratification and clinical decision-making [13,14]. Factors such as the severity of neutropenia, the duration and intensity of chemotherapy, and the implementation of prophylactic antimicrobial strategies all contribute to infection risk [15]. Additionally, the rise of antibiotic resistance further complicates treatment protocols, highlighting the need for continuous adaptation of antimicrobial strategies [16,17]. This study aims to investigate whether specific pediatric cancer types are associated with different risks or patterns of febrile neutropenia and sepsis [18]. By integrating real-time infection surveillance, resistance pattern analysis, and precision medicine approaches, this research seeks to contribute to a more tailored and effective management framework for pediatric oncology patients at risk of severe infections [19].

The results in this article are part of a larger research effort comparing two groups of pediatric oncology patients (febrile neutropenia vs. sepsis). Some variables, such as age, sex, ethnicity, residence, tumor-type distribution in G2, baseline neutrophil counts, and the median interval from chemotherapy to infection, have been previously reported in Cureus [20] and Farmacia [21]. However, the current manuscript explores a distinct research objective and applies a different analytical perspective, focusing specifically on the distribution of oncological diagnoses in relation to the clinical presentation and infection type.

## Materials and methods

Study design and setting

This retrospective study was conducted between 2018 and 2022 within the Hematology-Oncology Department of the Emergency Clinical Hospital for Children "Maria Sklodowska Curie" in Bucharest. We evaluated 150 cases of febrile neutropenia (FN) and sepsis recorded in 94 pediatric patients diagnosed with hematologic or oncologic disorders. Several patients experienced multiple infectious episodes throughout the study period, either of the same type or alternating between febrile neutropenia and sepsis, with either sequence occurring at different time points. Each episode was considered a distinct case if it was separated by a clinically resolved interval and required a new course of treatment. Accordingly, episodes were analyzed at the event level rather than clustered by patient, and no statistical adjustment for repeated measures was applied. Thus, a total of 150 cases were included. Data were collected from the hospital’s electronic database following the Ethics Committee's approval (Approval No. 34272/25.07.2024), in accordance with ethical research standards.

Patient selection and grouping criteria

Patients were assigned to one of two distinct groups based on clinical presentation and blood culture results obtained at the onset of each febrile episode. Clinicians were not formally blinded during this process; however, classification relied strictly on objective criteria. Group G1 (FN) included cases characterized by fever and negative blood cultures (n = 100), while group G2 (Sepsis) comprised cases with fever and positive blood cultures, confirming a bacterial infection (n = 50). It is important to note that the classification was applied per infectious episode rather than per individual patient, as multiple episodes could occur in the same patient. Group allocation was performed prospectively at the time of each episode, based on objective clinical and microbiological findings, not retrospectively based on subsequent evolution or laboratory trends.

Exclusion criteria

Exclusion criteria comprised cases in which blood cultures were performed solely to differentiate between hematologic and infectious conditions in the absence of clinical signs of infection. Also excluded were febrile episodes with negative blood cultures in which the etiology was identified as viral, as well as episodes with positive blood cultures where the infectious focus was clearly localized outside the bloodstream (e.g., respiratory or urinary tract infections).

Inclusion criteria and definitions

Patients were eligible for inclusion if they had a confirmed oncologic diagnosis and developed a febrile episode during or shortly after the administration of chemotherapy. Infectious episodes were included regardless of the ANC recorded at fever onset, provided that clinical signs of infection were evident and the episode resolved with antibiotic therapy.

In line with established clinical definitions, episodes with ANC < 500/mm³ were considered classical (numerical) neutropenia and inherently carried an elevated infectious risk [1]. However, emerging evidence and expert guidelines have recognized that ANC alone may not accurately reflect neutrophil competence in immunocompromised hosts [2-4]. Therefore, episodes with preserved or recovered ANC values were also included if the patient exhibited signs suggestive of immune dysfunction, either due to the underlying malignancy or recent exposure to cytotoxic chemotherapy [5,6].

This condition, termed functional neutropenia, reflects the state in which neutrophil function (e.g., chemotaxis, phagocytosis, and intracellular killing) remains impaired despite numerically adequate counts. Such dysfunction has been documented in pediatric oncology patients, particularly following granulocyte colony-stimulating factor (G-CSF) administration, where numerical recovery may precede true immunological restoration [3,5]. Clinical inclusion of these patients is consistent with international recommendations, including those of the Infectious Diseases Society of America (IDSA) and European Society for Medical Oncology (ESMO), which emphasize that both classical and functional neutropenia confer a similar risk for severe infections and justify equivalent therapeutic vigilance [1,4-7].

As such, the present study adopted a broadened inclusion strategy to reflect real-world risk stratification and to account for functional immune suppression beyond standard ANC thresholds.

Operational Definition of Sepsis

Sepsis was defined as a febrile episode with ≥1 positive blood culture and no alternative infectious focus (primary bloodstream infection). Single-positive cultures of common skin commensals were counted only when clinically adjudicated as significant (compatible presentation, inflammatory response, and no plausible alternative source) [4,22-24].

**Figure 1:**
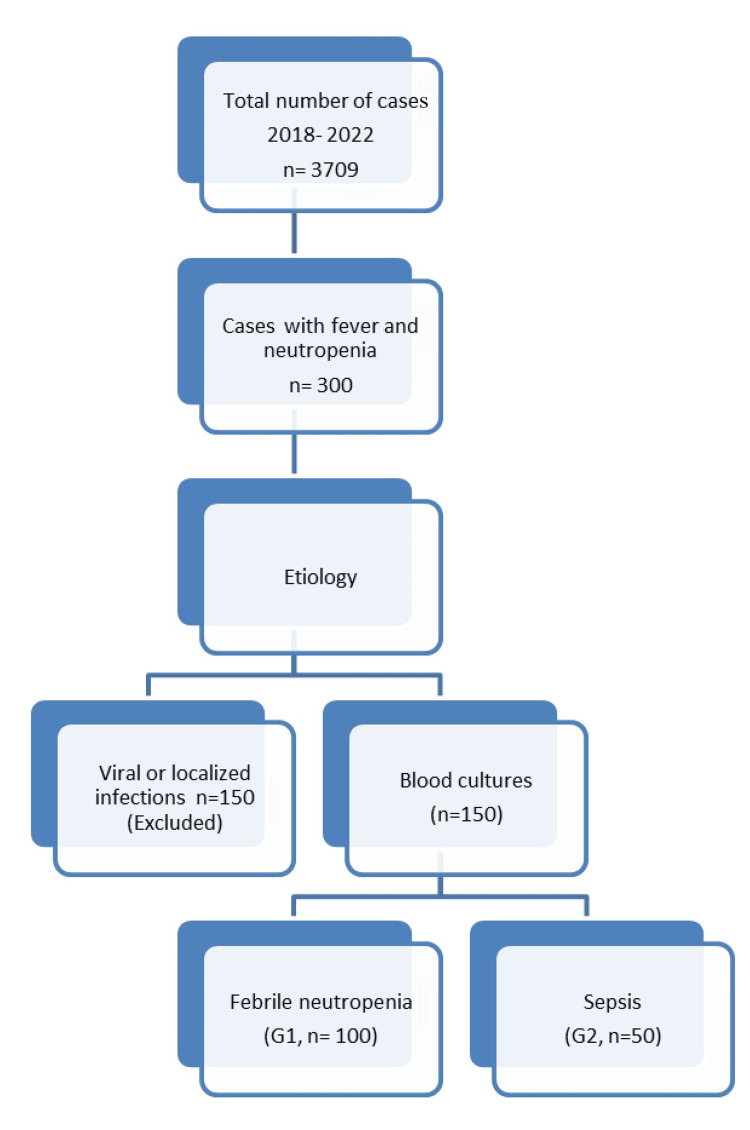
A decision flow diagram for case selection.

Figure 1 depicts the decision flow for case selection and per-episode allocation to G1 (FN) and G2 (sepsis).

Variables analyzed

The variables analyzed in this study included demographic, clinical, and oncologic data relevant to infection risk assessment. Demographic variables encompassed age at the time of infection, gender, place of residence, and ethnicity. Clinical data included anthropometric parameters such as weight, height, and body mass index, as well as personal medical history and family history of malignancy. Oncologic data included the specific tumor diagnosis and the interval between the last chemotherapy session and the onset of fever or bacteremia. To facilitate comparative analysis, oncologic diagnoses were grouped into the following categories: bone and soft tissue tumors, central nervous system (CNS) tumors, renal tumors, lymphomas, leukemias, hepatoblastomas, histiocytic disorders, and other tumor types.

Statistical analysis

Data were analyzed using Microsoft Excel 2021 (Microsoft Corporation, Redmond, WA) and IBM SPSS Statistics version 26.0 (IBM Corp., Armonk, NY). Normality of distribution for quantitative variables was assessed using the Kolmogorov-Smirnov and Shapiro-Wilk tests. For comparison of qualitative dichotomous variables, Fisher’s exact test and Pearson’s chi-square test were used.

Comparisons of means were performed using the independent-samples t-test, while the Mann-Whitney U test was applied for comparing medians of non-parametric variables. P-values were calculated using the independent-samples t-test for mean age comparison and chi-square test for categorical age group comparisons, with a statistically significant threshold of p < 0.05.

## Results

In the distribution by age group, the one to four years category was the most frequently represented in both cohorts. A notable proportion of patients also fell within the nine to 13 years interval. The mean age at infection differed significantly between the two groups, with values of 8.23 (± 2.6) years in group 1 and 5.25 (± 1.2) years in group 2 (p = 0.001). In group 1, the two aforementioned age brackets (1-4 and 9-13 years) each accounted for 28% of the patients. In contrast, group 2 had a clear predominance of children aged one to four years (56%), a difference that reached statistical significance (p = 0.01). The lowest representation in both groups was observed among infants under one year of age (9% in group 1 vs. 4% in group 2). These findings are summarized in Table 1.

**Table 1:** Age group distribution. Data for continuous variables are presented as mean ± standard deviation (SD). Categorical variables are shown as counts (percentages). An independent samples t-test was used for comparing mean age (t (147) = 9.60). Chi-square tests were used for comparing each age group category individually. A p-value of <0.05 was considered statistically significant. Previously published data [20].

Parameter	Group 1 (n = 100)	Group 2 (n = 50)	p-value (test)
Mean age (years)	8.23 ± 2.6	5.25 ± 1.2	p = 0.001; t(147) = 9.60
Under 1 year	9 (9%)	2 (4%)	p = 0.438; χ² = 0.60
1–4 years	28 (28%)	28 (56%)	p = 0.002; χ² = 10.01
5–8 years	14 (14%)	7 (14%)	p = 1.000; χ² = 0.00
9–13 years	28 (28%)	8 (16%)	p = 0.156; χ² = 2.01
Over 14 years	21 (21%)	5 (10%)	p = 0.147; χ² = 2.10

Table 2 summarizes the demographic characteristics of the two groups. No statistically significant differences were found in terms of gender, residential area, or ethnicity. Nevertheless, male patients were more frequent in group 1 (65%) compared to group 2 (56%), although this difference was not statistically significant (p = 0.28). Both groups exhibited a relatively balanced distribution regarding place of residence.

**Table 2:** Demographic characteristics of the two groups. Data are presented as counts (percentages). Chi-square tests were applied both at the grouped level and for individual categories to assess demographic differences between group 1 and group 2. Global and individual p-values (a p-value of <0.05 is considered statistically significant), along with χ² statistics, are reported. A p-value of <0.05 was considered statistically significant. 95% confidence intervals estimated by Monte Carlo simulation are reported. Previously published data [20].

Parameter	Group 1 (n = 100)	Group 2 (n = 50)	Statistical test result
Gender			Global: p = 0.28; χ² = 0.80; 95% CI (Monte Carlo): 0.14-0.58
Male	65 (65%)	28 (56%)	p = 0.372; χ² = 0.80
Female	35 (35%)	22 (44%)	p = 0.372; χ² = 0.80
Environment			Global: p = 0.56; χ² = 0.16; 95%CI (Monte Carlo): 0.22-0.81
Rural	51 (51%)	23 (46%)	p = 0.686; χ² = 0.16
Urban	49 (49%)	27 (54%)	p = 0.686; χ² = 0.16
Ethnicity			Global: p = 0.90; χ² = 0.07; 95%CI (Monte Carlo): 0.78 - 1.00
Romanian	87 (87%)	45 (90%)	p = 0.790; χ² = 0.07
Other	13 (13%)	5 (10%)	p = 0.790; χ² = 0.07

When accounting for median age, the oldest patients were encountered in the histiocytic disorders (median age = 15 (15-16) years), leukemias (median age = 8 (1-17) years), and lymphomas (median age = 7 (3-11) years), respectively. Statistically significant differences were noticed between the two groups regarding the bone and soft tissue tumors (p = 0.04) and leukemias (p = 0.02). The earliest onset age was noticed in CNS tumors (median age = 1 (0.5-4) year), renal tumors (median age = 1 (0.5-7) year), and hepatoblastomas (median age = 0.75 (0.33-1) year). When accounting for all tumors, the difference was statistically significant regarding the median age (p = 0.001). Data are synthesized in Table 3.

**Table 3:** Median age based on group and tumor types. "Other" includes rare pediatric malignancies not represented in the predefined diagnostic categories, such as tumors with atypical localization or undefined histology. Data are presented as median and range. The Mann–Whitney U test was used to compare the median age at diagnosis between group 1 and group 2 for each oncological category. For the comparison of median age across all tumor types in the total cohort (n = 150), the Kruskal–Wallis H test was applied. Two-tailed p-values were reported, with statistical significance set at p < 0.05.

Oncological diagnoses	Group 1 (n = 100)	Group 2 (n = 50)	P-value between groups	Total (n = 150)	P-value within total
Bone and soft tissue tumors	6 (2 - 8)	4 (2 - 10)	0.04	4 (2 - 10)	0.001
CNS tumors	1 (0.5-4)	1 (0.5 - 4)	1	1 (0.5 - 4)	
Renal tumors	2 (0.5 - 6)	1 (0.5 - 7)	0.84	1 (0.5 - 7)	
Lymphomas	8 (4 - 11)	6 (3 - 9)	0.34	7 (3 - 11)	
Leukemias	9 (2 - 17)	7 (1 - 14)	0.02	8 (1 - 17)	
Hepatoblastomas	0.66 (0.33 - 1)	0.75 (0.75 - 0.75)	0.98	0.75 (0.33 - 1)	
Histiocytic disorders	15 (0.25 - 16)	15 (15 - 16)	0.87	15 (15 - 16)	
Other	3 (1 - 9)	4 ( 1 - 8)	0.12	4 (1 - 9)	

Frequency and distribution of groups based on oncological cases are highlighted in Table 4. The majority of cases were bone and soft tissue tumors (28%/42) as well as leukemias (22.7%/34). Although there were no statistically significant differences between the tumor type distribution for most of the tumors, a statistically significant difference regarding the frequency of CNS tumors was highlighted, with a higher frequency being reported in group 2 (8% vs. 20%/8 vs. 10, p = 0.03) [21].

**Table 4:** Frequency and distribution of oncological diagnoses based on groups. P-values were calculated using Pearson’s chi-square test to compare tumor type distribution between the two groups. "Other" includes rare pediatric malignancies not represented in the predefined diagnostic categories, such as tumors with atypical localization or undefined histology. 95% confidence intervals estimated by Monte Carlo simulation are reported. Previously published data [21].

Oncological diagnoses	Group 1 (n = 100)	Group 2 (n = 50)	Total (n = 150)	P-value	95% CI (Monte Carlo)
Bone and soft tissue tumors	31 (31%)	11 (22%)	42 (28%)	0.07	0.01 – 0.34
CNS tumors	8 (8%)	10 (20%)	18 (12%)	0.03	0.01 – 0.19
Renal tumors	9 (9%)	5 (10%)	14 (9.3%)	0.84	0.52 – 0.98
Lymphomas	18 (18%)	6 (12%)	24 (16%)	0.34	0.21 – 0.70
Leukemias	20 (20%)	14 (28%)	34 (22.7%)	0.26	0.19 – 0.55
Hepatoblastomas	4 (4%)	1 (2%)	5 (3.3%)	0.52	0.28 – 0.74
Histiocytic disorders	5 (5%)	0 (0%)	5 (3.3%)	0.28	0.11 – 0.61
Other	5 (5%)	3 (6%)	8 (5.4%)	0.84	0.55 – 1.00

**Figure 2:**
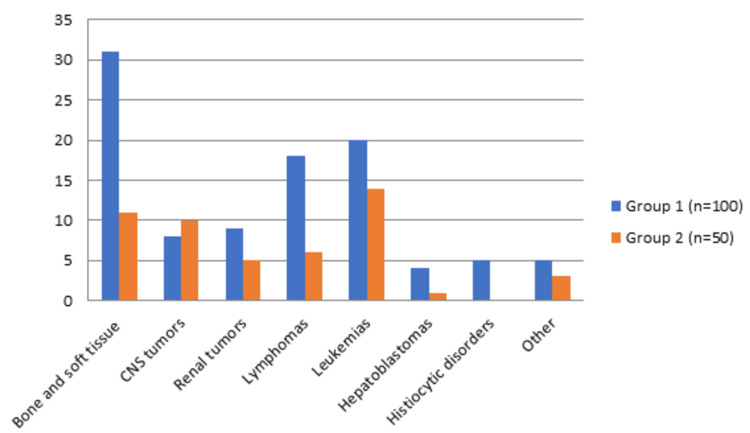
Distribution of diagnoses across groups (G1 vs. G2).

To aid interpretation, Figure 2 presents a bar chart of the frequency and distribution of oncological diagnoses across G1 and G2, corresponding to the table above.

Details on the median duration from the initiation of oncological treatment to the onset of febrile episodes are presented in Table 5. The median duration varied significantly across different tumor types, ranging from two to 19 days (p = 0.02). The longest durations were observed in patients with lymphomas (17 days) and leukemias (19 days), whereas the shortest duration was recorded in patients with hepatoblastoma (two days) [21].

**Table 5:** Median interval from treatment to infection. P-value calculated using the Kruskal-Wallis test. "Other" includes rare pediatric malignancies not represented in the predefined diagnostic categories, such as tumors with atypical localization or undefined histology. 95% confidence intervals estimated by Monte Carlo simulation are reported. Previously published data [21].

Parameters	Median interval from treatment to infection (days)	P-value + 95% CI
Bone and soft tissue tumors	11.5 (8.5 - 20)	0.02 (0.01 – 0.08)
CNS tumors	15 (8.5 - 36.75)	
Renal tumors	14 (6.5 - 23.75)	
Leukemias	19 (13 - 38)	
Lymphomas	17 (11.75 - 34)	
Hepatoblastomas	2 (1 - 12)	
Histiocytic disorders	12 (1.5 - 78)	
Other	11.5 (6.25 - 16.5)	

Table 6 presents the mean values of leukocytes, neutrophils, and C-reactive protein (CRP) at admission, stratified by oncological diagnosis. Statistically significant differences were observed for all markers (p = 0.001). These findings are detailed in Table 6.

**Table 6:** Comparison of mean leukocyte, CRP, and neutrophil values at infection according to oncological diagnosis. P-value calculated using the Kruskal-Wallis. "Other" includes rare pediatric malignancies not represented in the predefined diagnostic categories, such as tumors with atypical localization or undefined histology.

Oncological diagnoses	Mean leukocytes at infection	Mean CRP at infection	Mean neutrophils at infection
Bone and soft tissue tumors	8.72 (±1.19)	38.19 (±3.19)	6.11 (±1.06)
CNS tumors	9.11 (±2.32)	44.82 (±5.19)	7.45 (±1.89)
Renal tumors	9.45 (±1.65)	41.19 (±6.21)	6.38 (±1.19)
Lymphomas	10.82 (±1.73)	51.12 (±5.41)	7.69 (±1.55)
Leukemias	11.45 (±2.09)	46.19 (±6.12)	8.12 (±2.01)
Hepatoblastomas	7.19 (±1.09)	33.17 (±1.91)	5.49 (±0.85)
Histiocytic disorders	7.24 (±0.93)	34.18 (±1.89)	5.83 (±1.01)
Other	8.28 (±1.79)	40.19 (±5.17)	6.02 (±1.86)
P-value	0.001	0.001	0.001

Table 7 presents the median neutrophil counts at infection onset across oncological diagnostic groups. Significant variation was observed, with the lowest values in lymphomas and leukemias and the highest in histiocytic disorders (p = 0.003).

**Table 7:** Median neutrophil values at infection onset during febrile episode admission by oncological diagnostic groups. P-value calculated using the Kruskal-Wallis. "Other" includes rare pediatric malignancies not represented in the predefined diagnostic categories, such as tumors with atypical localization or undefined histology.

Oncological diagnoses	Median value	P-value
Bone and soft tissue tumors	0.94 (0.02 - 6.82)	0.003
CNS tumors	1.23 (0.06 - 6.76)	
Renal tumors	3.26 (2.3 - 7.29)	
Lymphomas	0.18 (0.02 - 3.34)	
Leukemias	0.27 (0.02 - 1.32)	
Hepatoblastomas	0.28 (0.08 - 2.99)	
Histiocytic disorders	5.85 (1.59 - 9.38)	
Other	4.25 (0.48 - 9.91)	

## Discussion

The findings of this study highlight significant associations between oncological diagnoses and the occurrence of FN and sepsis in pediatric patients [1,5]. The distribution of tumor types within the study population indicates that hematologic malignancies, particularly leukemias and lymphomas, are major contributors to infection risk. This pattern is well-documented in the literature, as these malignancies are commonly associated with repeated exposure to cytotoxic regimens known to induce profound and sustained immunosuppression, affecting both innate and adaptive arms of the immune system [6,10]. Our results are in line with previous studies demonstrating that patients with acute myeloid leukemia (AML) and acute lymphoblastic leukemia (ALL) experience more severe and prolonged episodes of neutropenia, thereby increasing their vulnerability to severe infectious complications [2,6,9]. The higher incidence of FN and sepsis in leukemia patients compared to those with solid tumors reinforces the need for tailored infection monitoring and targeted antimicrobial strategies in this subgroup [4,7,13].

A statistically significant difference regarding the age of onset between the two groups was highlighted in patients with leukemia and bone and soft tissue tumors, with sepsis being highlighted in younger patients in both cases. This particularity can emphasize that younger patients can be more susceptible to sepsis in these two cases; there is a notable difference between the two groups regarding the age of onset of all malignancies. Further studies should highlight the particularities of these two pathologies that may involve different immune pathways, as well as a certain weakening of the immune system, especially in leukemia patients.

Beyond hematologic malignancies, our study also identified a statistically significant correlation between CNS tumors and an increased incidence of bloodstream infections. A higher proportion of CNS tumor cases was observed in the sepsis group compared to the FN group [3,12]. This pattern may be explained by several factors specific to CNS tumor management, including chronic corticosteroid administration, frequent surgical interventions, and the prolonged presence of central venous catheters. These elements may cumulatively impair the host’s capacity to mount an effective immune response. Such findings are in agreement with existing literature highlighting the heightened infection risk in pediatric patients with solid tumors, particularly those undergoing multimodal therapy involving surgery, radiation, and myelosuppressive chemotherapy [8,14,15].

The median time from initiation of chemotherapy to the onset of febrile episodes also varied by tumor type. Patients diagnosed with leukemia and lymphoma had the longest intervals between treatment and infection onset, which may reflect the delayed nadir of neutropenia typically seen with intensive regimens. Conversely, patients with hepatoblastoma exhibited a notably shorter interval to fever onset, potentially due to cumulative marrow toxicity from dense chemotherapy protocols. These differences support earlier studies emphasizing the influence of treatment timing, intensity, and hematologic dynamics on infection susceptibility [1].

In addition to temporal differences in fever onset, our data revealed statistically significant variations in inflammatory markers at admission across tumor types. Leukocyte and CRP levels were notably elevated in patients diagnosed with hematological malignancies, particularly leukemias and lymphomas. These findings may reflect both the underlying immunopathology of the malignancy and the systemic inflammatory milieu triggered by tumor burden or infection. Conversely, patients with solid tumors such as hepatoblastomas and histiocytic disorders demonstrated lower levels of these markers, which could indicate a more attenuated inflammatory response or differences in the host's immune reserve following treatment. The observed patterns support previous evidence that tumor biology influences the host’s immunologic profile, potentially affecting the intensity and detectability of febrile episodes [22]. These results underline the importance of integrating diagnosis-specific inflammatory profiles into infection risk stratification models.

Our analysis also revealed significant variation in neutrophil counts at the time of fever onset among tumor types. The lowest median neutrophil levels were observed in patients with hepatoblastomas, suggesting that the depth of neutropenia remains a critical predictor of infection risk. This observation underscores the relevance of continuous monitoring of hematologic parameters and supports the inclusion of prophylactic strategies, such as antibiotic prophylaxis and G-CSF support, in treatment protocols for patients at higher infectious risk [5,6,8].

However, neutrophil recovery following G-CSF administration does not guarantee restored immune competence. Several studies have shown that, even when ANC returns to normal levels, neutrophil functionality remains impaired. Essential functions such as chemotaxis, intracellular bactericidal activity, oxidative burst capacity, and pathogen recognition may be suppressed for days or even weeks after chemotherapy [23-28]. These qualitative deficits are not always detectable through standard hematologic assessments but may substantially affect the patient’s ability to respond to pathogens, resulting in a risk profile similar to that of profound neutropenia.

In this context, we adopted a broadened inclusion strategy, extending our analysis to patients who presented with clinical signs of infection during or shortly after chemotherapy, regardless of their absolute neutrophil count. This decision reflects the concept of functional neutropenia, a condition in which neutrophil numbers are preserved but their functional capacity is significantly compromised [26]. While this phenotype is often underreported and excluded from traditional FN research, there is growing support for its clinical relevance, particularly in pediatric oncology settings where patients undergo repeated cycles of intensive therapy [19-21,23-28]. Recognizing functional neutropenia allows for a more comprehensive evaluation of infection risk and may improve risk stratification, resource allocation, and clinical outcomes. Future research should aim to develop and validate simple clinical markers of functional neutropenia, such as neutrophil oxidative burst or chemotaxis tests, that could be integrated into pediatric oncology practice. These tools may support better identification of vulnerable patients and enable more personalized infection risk management.

Moreover, while tumor type remains a core determinant of infectious vulnerability, our findings suggest that infection risk is shaped by a multifactorial interplay of host-related and treatment-related factors. Age, comorbidities, cumulative chemotherapy exposure, nutritional status, and local care practices (e.g., catheter maintenance) may all modulate susceptibility to infection and impact the clinical course of febrile episodes [1,12,18]. These insights call for the development of predictive models that integrate not only tumor biology and chemotherapy schedules but also dynamic clinical parameters and individual patient profiles. Such models may guide clinicians in identifying patients who require more intensive surveillance, earlier empirical antibiotic treatment, or tailored prophylactic regimens. In this context, residential background (urban vs. rural) was also examined as an exploratory factor, acknowledging its potential, albeit untested, influence on infection risk and access to timely medical care.

Finally, the wide variability in neutrophil counts observed at fever onset, depending on cancer type and treatment phase, reinforces the importance of individualized monitoring. Standardized thresholds for FN may fail to capture at-risk patients with functional immunosuppression, potentially delaying interventions. Integrating real-time hematologic, clinical, and biochemical data into patient care protocols may help clinicians anticipate complications and deliver more timely, effective infection management strategies [7,11,17].

This study has several limitations that warrant consideration. One important limitation, also frequently noted in the literature, is the relatively low number of cases recorded during the study period, a factor that may have been influenced by the restrictive measures implemented during the COVID-19 pandemic (2020-2022) [29]. Since a formal power analysis was not conducted prior to study initiation, the sample size and statistical power were estimated based on feasibility considerations, which may be viewed as an additional methodological limitation. Furthermore, as this study was conducted retrospectively in a single tertiary pediatric oncology center, its findings may not be fully generalizable to other clinical settings with different patient profiles, therapeutic protocols, or infection control strategies. In addition, pediatric severity and early warning scoring systems, such as Pediatric Early Warning Score (PEWS) or Sequential Organ Failure Assessment (SOFA), were not applied, as these are not part of routine clinical practice in our center. For the same reasons, Sepsis-3 pediatric (Phoenix) criteria could not be applied, which may limit severity stratification and comparability with studies using consensus-based definitions. Moreover, no formal blinding of clinicians or inter-rater reliability checks were applied during episode classification. Although classification was based on objective and consistently applied criteria, the absence of blinding may have introduced a degree of classification bias.

A further limitation relates to patients who experienced more than one infectious episode during the study period. Although each episode was considered distinct only when clinically and temporally separated, no specific statistical adjustment for repeated measures within the same individual was applied. We recognize that this may introduce a degree of intra-patient correlation; however, the overall frequency of recurrence was limited, and we do not believe it substantially impacted the interpretation of results. Additionally, oncological diagnoses were categorized into broader groups to ensure adequate statistical power, given the low frequency of certain malignancies. However, this approach may have obscured clinically relevant differences between specific subtypes, such as ALL versus AML or medulloblastoma versus glioma, which should be addressed in future subtype-level analyses. While this approach facilitated comparative analysis, it may have concealed specific infection trends associated with less common tumor types. Nevertheless, the study offers valuable insights into infection risk stratification based on oncological diagnosis, underlining the importance of addressing both classical and functional neutropenia in clinical assessments. Prospective multicenter studies are needed to confirm these findings and to inform the development of more refined and comprehensive predictive models.

## Conclusions

This study highlights the considerable variability in the risk of febrile neutropenia and sepsis among pediatric oncology patients, depending on the type of malignancy. Hematologic cancers, particularly leukemias and lymphomas, were associated with longer durations of neutropenia and a higher incidence of febrile episodes, warranting intensified infection monitoring in these subgroups. The observed differences in the timing and frequency of febrile episodes across tumor types emphasize the need for individualized risk assessment. Recognizing these patterns enables clinicians to better anticipate infection-related complications and to tailor prophylactic interventions accordingly.

Overall, our findings support a more personalized approach to infection surveillance and management in pediatric oncology, contributing to improved outcomes through timely and targeted strategies for patients with febrile neutropenia and sepsis. Moreover, the integration of tumor-specific inflammatory profiles, timing of infection onset, and recognition of functional neutropenia may enhance current risk stratification models and optimize therapeutic decision-making. In pediatric oncology, these findings suggest that infection monitoring protocols may benefit from being adapted according to diagnosis type, supporting more personalized approaches in future clinical practice.
